# Ogilvie Syndrome and Acute Kidney Injury: A Rare Complication of Cesarean Section and Preeclampsia

**DOI:** 10.3390/jcm12062249

**Published:** 2023-03-14

**Authors:** Maria Rita Stancanelli, Linda Njandjo, Federica Errigo, Antioco Fois, Domenico Santoro, Giorgina Barbara Piccoli, Massimo Torreggiani

**Affiliations:** 1 Unit of Nephrology and Dialysis, Department of Clinical and Experimental Medicine, A.O.U. “G. Martino”, University of Messina, 98125 Messina, Italy; 2 Néphrologie et Dialyse, Centre Hospitalier Le Mans, 72037 Le Mans, France; 3 Nephrology Unit, Department of Translational and Precision Medicine, Sapienza University of Rome, 00185 Rome, Italy

**Keywords:** paralytic ileus, acute colonic pseudo-obstruction (ACPO), pregnancy, malaria, complication, hypertensive disorders of pregnancy

## Abstract

Ogilvie syndrome, or acute colonic pseudo-obstruction (ACPS) is a rare occurrence, usually following surgery. It consists of a massive dilatation of the cecum, whose diameter becomes greater than 10 cm; its severity is variable, but, if not promptly recognized, it may be life-threatening. Acute kidney injury (AKI) is reported in this context due to both septic complications and to effective hypovolemia. ACPS most commonly affects males and individuals older than 60. In women, the median age at diagnosis is lower due to a strong association with Caesarean sections. The differential diagnosis after delivery may be challenging, due to a potential overlap of symptoms with preeclampsia or hemolysis low platelet elevated liver enzymes (HELLP) syndrome, both associated with AKI. The case herein discussed, regarding a 35-year-old woman, who developed AKI and Ogilvie syndrome after a Caesarean section for preeclampsia, may exemplify these diagnostic and therapeutic challenges, and is intended to raise awareness on this unusual complication of Caesarean delivery.

## 1. Background

Due to the protean presentation of the different hypertensive disorders of pregnancy, and to their high prevalence (up to 10% of pregnancies, considering the whole spectrum, from pregnancy induced hypertension to preeclampsia to hemolysis low platelet elevated liver enzymes (HELLP) syndrome), the differential diagnosis of other complications of pregnancy and of concomitant diseases may be difficult [1,2].

The case herein discussed is a good example of this complexity, and reports on an unusual condition, Ogilvie syndrome or acute colonic pseudo-obstruction (ACPO), that may arise as a complication of surgery, including Caesarean section, and, as in this case, may lead to acute kidney injury (AKI), thus calling for a differential diagnosis with renal involvement in the course of preeclampsia or HELLP syndrome [3,4,5].

Ogilvie syndrome, is characterized by massive dilatation of the cecum, reaching a diameter greater than 10 cm, in the absence of mechanical obstruction [3,4,5]. The dilatation may be limited to the caecum or involve other intestinal segments, up to the entire colon [3,4,5]. Symptoms are related to abdominal pain and distension, nausea, and vomiting may be of variable severity. The incidence is estimated at about 100 cases per 100,000 hospital admissions every year [5]. The prevalence is higher after the age of 60, however, in women, it occurs at a lower mean age since it shows an incidence peak related to Caesarean sections [4,5].

Due to the frequent subacute onset, the differential diagnosis includes various causes of abdominal pain and, after surgery or delivery, diagnostic delays are common [6]. Obesity is a risk factor for APCO syndrome and other complications of surgery, as very well acknowledged in kidney transplantation [7].

The case reported herein regards a patient who developed ACPO following Caesarean section for preeclampsia and HELLP syndrome; the discussion of the diagnostic challenges may help increasing awareness in this unusual complication of a complicated pregnancy.

### Case Report

A 35-year-old woman of African origin (Guinea Conakry) was referred during her pregnancy to a tertiary care obstetrical center. Her medical history was significant for malaria infection, allergy to quinine, and no surgery other than appendectomy during her adolescence. She had no family members affected by kidney diseases nor with history of preeclampsia. Her mother has hypertension.

Her obstetric history (G10P3) was complex. She experienced:

– three ectopic pregnancies at the age of 21, 23, and 24;
– one twin pregnancy at the age of 25, complicated by preeclampsia;
– three miscarriages at the age of 26, 27, and 28;
– one successful pregnancy at the age of 29, without complications;
– one miscarriage at the age of 32.

The 10th pregnancy we are referring to occurred at the age of 35 (spontaneous pregnancy). At the start of pregnancy her body weight was 110 kg with a body mass index of 41.9 kg/m^2^. She was on therapy with nicardipine 50 mg twice a day, and labetalol 200 mg one a day for chronic hypertension.

At the thirty-first week of gestation, she presented with fever (38.7 °C), tinnitus, and emesis. Laboratory tests showed: hemoglobin 10.4 g/dL, leukocytes 12 g/L, platelets 83 g/L, CRP 243 mg/L, fibrinogen > 7 g/L, haptoglobin < 10 g/L, spot proteinuria 3.89 g/L, proteinuria/creatininuria ratio 1.16 g/g, ASAT 53 IU/L, ALAT 25 IU/L, bilirubin 34 µmol/L, creatinine 40 µmol/L, sodium 135 mmol/L, and potassium 3 mmol/L. Upon physical examination, lower limbs edema, normal blood pressure, absence of headache, and visual disturbances were reported; her body weight was 130 Kg. On the account of a recent trip to her home country, malaria was suspected and confirmed by 20% parasitemia on peripheral blood smear, with numerous trophoblasts of Plasmodium falciparum.

Furthermore, due to the presence of intense proteinuria, unexplained by the malaria crisis, preeclampsia superimposed on chronic hypertension was suspected. She was treated with artesunate, magnesium sulphate, and one unit of platelet concentrate was transfused. Following neurological deterioration (Glasgow Coma Scale 13/15) an emergency Caesarean section was performed, with the birth of a healthy male baby weighing 1700 g (68.69 centile). The baby was transferred to the neonatal intensive care unit for surveillance, he never required intubation, and was discharged 24 days later in good clinical condition and a body weight of 2220 g.

After delivery, the mother’s neurological picture worsened. Computed tomography and transcranial Doppler ultrasound excluded acute brain lesions, cerebral hypoperfusion, and posterior reversible encephalopathy syndrome. Hemolytic anemia persisted (hemoglobin 9.8 g/dL, haptoglobin < 10 g/L) and jaundice developed, with a total bilirubin of 90 µmol/L. Therefore, the patient was transferred to the intensive care unit (ICU). During the first night in the ICU, fever peaked at 38.6 °C, the abdomen was distended and painful. An abdominal CT scan revealed “postoperative ileus and uterine hyperemia” leading to a suspicion of endometritis, on whose account antibiotic therapy with metronidazole and meropenem was started. In the following hours, she developed oliguria and progressive kidney function impairment, reaching a level of serum creatinine of 210 µmol/L.

**Figure 1:**
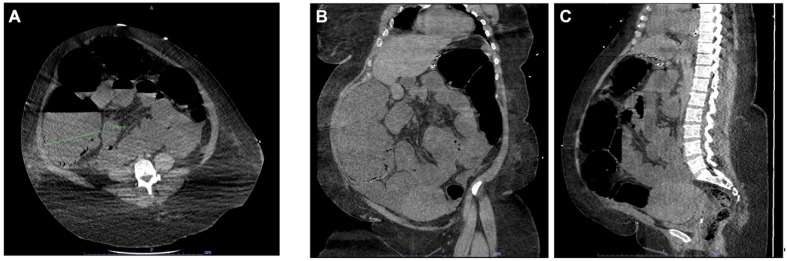
(**A**) Abdominal CT scan axial section showing the massive dilation of the caecum; (**B**) CT scan coronal section showing the dilation of the entire colon; (**C**) sagittal view.

While diuresis partially responded to saline infusion, pain and abdominal distension persisted. A second CT scan, repeated three days later, showed massive colon dilation with a cecum measuring 115 mm in diameter (Figure 1). After a failed attempt of colonoscopy decompression, in the absence of perforation and in the presence of initial signs of hypovolemic shock, she underwent subtotal colectomy and ileostomy. Creatinine peaked to 242 µmol/L after surgery, with progressive normalization in the following days.

Four months after delivery, the clinical conditions improved, blood pressure control was good at the 24 h monitoring, on labetalol 200 mg twice daily, ramipril 5 mg, and nicardipine 50 mg. Kidney function was normal (creatinine 64 µmol/L, eGFR according to CKD-EPI formula 107 mL/L min/1.73 m^2^, proteinuria 0.119 g/24 h, and microalbuminuria 7 mg/24 h), anemia and thrombocytopenia resolved and liver enzymes normalized.

According to the patient’s desire, six months after delivery an ileo-rectal anastomosis was created and digestive continuity was restored. The patient’s body weight was 114 kg at the time of the intervention.

At the last follow-up, fourteen months after delivery, serum creatinine was 73 µmol/L (eGFR CKD-EPI 91.4 mL/min/1.73 m^2^), with spot proteinuria < 6 mg/dL. Blood pressure control was good after further modulation of the therapy, now including spironolactone 50 mg, ramipril 5 mg, and nicardipine 50 mg; a weight loss program was started.

## 2. Discussion

When a patient experiences a complication during her pregnancy, due to the protean manifestations associated with preeclampsia or HELLP syndrome, these conditions are usually the first considered in the differential diagnosis; however, this may delay the correct diagnosis, as our case exemplifies.

In the context of a highly complex clinical situation, such as the one of our patient who presented PE-HELLP, severe malaria, fever, and abdominal pain and distension, ascribing abdominal pain to surgery was legitimate. Furthermore, fever could be caused by malaria, and AKI, presumably due to abdominal sequestration and relative hypovolemia, was interpreted in the context of an HELLP syndrome; to increase the difficulties, platelet count was low, probably due to sepsis, and hemolysis could be both the result of a malaria crisis or HELLP. The overlap and the differences between the four conditions are reported in Table 1 [1,2,3,4,5,8,9].

Fever, initially explained by malaria, was indeed a hallmark of the Ogilvie syndrome, since, as the pressure on the intestinal wall increases, there is a translocation of fluids and bacteria up to colonic perforation and peritonitis [3,4,5].

However, the massive abdominal distension and the increasing pain unresponsive to antibiotics pointed to an alternative diagnosis. However, even if the relationship between Ogilvie’s syndrome and Caesarean section is described, the intestinal dilatation, already present at the first scan, was initially overlooked.

A systematic review published in 2017 retrieved 125 cases of postpartum Ogilvie syndrome published since 2002, of which, 62% occurred after Caesarean delivery. In 22% of these cases, the indication for Caesarean delivery was preeclampsia or HELLP syndrome; diagnosis was delayed in about one-third of the cases [6]. Obesity, as in the case of our patient, is also a risk factor for developing Ogilvie syndrome. As reported in a study on patients with kidney transplantation, a body mass index >30 kg/m^2^ represents an important risk factor, probably related to mesenteric atherosclerosis and ischemia [7].

Postpartum Ogilvie syndrome may be more frequent than previously expected. A retrospective study analyzing cases of postpartum intestinal obstruction estimated an incidence of 1 in 1500 deliveries [10,11,12,13,14].

Early diagnosis is essential to initiate medical therapy with neostigmine or perform endoscopic decompression in order to avoid colectomy because intestinal perforation, reported in 30–60% of the pregnancy-related cases, requires laparotomy, and is associated with high mortality [6,10,11,12,13,14]. It is worth mentioning that colonoscopy seems superior to neostigmine treatment [15].

While Ogilvie syndrome has been reported in several infectious diseases, including COVID-19, one case only was reported in the context of severe malaria, even if the pathophysiology remains unknown [16,17].

AKI may complicate an Ogilvie syndrome. Abdominal distension leads to an increase in abdominal pressure and causes compartment syndrome resulting in hypovolemia and pre-renal AKI. The effect may be multiplied by sepsis [18,19]. Although we cannot completely exclude other causes of AKI, such as sepsis, malaria infection, or HELLP, the timeline of events makes ACOPS the most likely cause in our case.

In conclusion, Ogilvie syndrome should be kept in mind in the differential diagnosis of AKI after a Caesarean section, considering the increase in complicated pregnancies and Caesarean deliveries over the last years, because the consequences of a late diagnosis may be severe, and even life-threatening.

## Figures and Tables

**Table 1:** Differential diagnosis between Ogilvie syndrome, severe malaria, preeclampsia and hemolysis, elevated liver enzymes, and low platelets (HELLP) syndrome.

	Ogilvie Syndrome	Severe Malaria	Preeclampsia	HELLP
Fever	+++	+++	−	−
Abdominal pain	+++	+++	+	++
Headache	−	+	++	++
GI symptoms	+++	+	+/−	+/−
Swelling arms/legs	−	−	+/−	+/−
Visual impairment-scotomata	−	−	+/−	+/−
Weight gain	−/+	−	++	++
Hypertension	−	−	+++	++
proteinuria	−	−	++	++
Microhematuria	−	+/−	−	−
Neurological worsening	+/−	+++	−	++
Dyspnea/ARDS	+/−	+++	+/−	+/−
Hemolytic anemia	++	+++	+/−	+++
Thrombocytopenia	++	++	−	+++
AKI	++	+++	++	++
Increased bilirubin	++	+++	−	+/−
Liver enzymes	−	−	+/−	+++
Hyperlactatemia	−	+++	−	−
Hypoglycemia	−	++	−	−

GI: gastrointestinal; AKI: acute kidney injury.

## Data Availability

The data presented in this study are available on request from the corresponding author.
